# On-Chip Miniaturized Bandpass Filter Using GaAs-Based Integrated Passive Device Technology For L-Band Application

**DOI:** 10.3390/ma12183045

**Published:** 2019-09-19

**Authors:** Bao-Hua Zhu, Nam-Young Kim, Zhi-Ji Wang, Eun-Seong Kim

**Affiliations:** RFIC Center, Kwangwoon University, 447-1 Wolgye-Dong, Nowon-Ku, Seoul 139-701, Korea; zhuwangwhy@hotmail.com (B.-H.Z.); zhiji-wang@hotmail.com (Z.-J.W.)

**Keywords:** Bandpass filter (BPF), controllability, equivalent circuit model, GaAs-based integrated passive device (IPD)

## Abstract

In this work, a miniaturized bandpass filter (BPF) constructed of two spiral intertwined inductors and a central capacitor, with several interdigital structures, was designed and fabricated using integrated passive device (IPD) technology on a GaAs wafer. Five air-bridge structures were introduced to enhance the mutual inductive effect and form the differential geometry of the outer inductors. In addition, the design of the differential inductor combined with the centrally embedded capacitor results in a compact construction with the overall size of 0.037*λ*_0_ × 0.019*λ*_0_ (1537.7 × 800 μm^2^) where *λ*_0_ is the wavelength of the central frequency. For the accuracy evolution of the equivalent circuit, the frequency-dependent lumped elements of the proposed BPF was analyzed and modeled through the segment method, mutual inductance approach, and simulated scattering parameters (S-parameters). Afterward, the BPF was fabricated using GaAs-based IPD technology and a 16-step manufacture flow was accounted for in detail. Finally, the fabricated BPF was wire-bonded with Au wires and packaged onto a printed circuit board for radio-frequency performance measurements. The measured results indicate that the implemented BPF possesses a center frequency operating at 2 GHz with the insertion losses of 0.38 dB and the return losses of 40 dB, respectively, and an ultrawide passband was achieved with a 3-dB fraction bandwidth of 72.53%, as well. In addition, a transmission zero is located at 5.32 GHz. Moreover, the variation of the resonant frequency with different inductor turns and metal thicknesses was analyzed through the simulation results, demonstrating good controllability of the proposed BPF.

## 1. Introduction

With the rapid development of modern intelligence, communication and telecommunication modules are widely applied to all parts of daily life, known as “connecting everything”. The increasing demands of communication modules drive the rapid evolution of the passive components that make up these modules as well, such as a balun, power divider, filter, etc. A bandpass filter (BPF) is a device that allows signals of a certain frequency in the range of *f_a_*–*f_b_* to pass through as is, while others that are out of the range are restrained. Thus, the BPF is a vital passive device in the radio frequency (RF) front-end module of a communication system, and its properties affect the performance of the overall operating system [1,2]. Therefore, the design and fabrication technology are attractive for researchers with the evolution of RF/microwave engineering.

Many works regarding BPF with various manufacturing techniques have been presented in recent years. Polymer substrate-based microstrip BPFs, patterned by the lamination technique and wet chemical etching process, have been reported [3,4]. The flexible multi-passband design approach, low fabrication cost, and simple manufacturing process are obtained via the stub-loaded resonator, step impedance resonator, coupling methodology, and the application of a low-cost substrate. Nevertheless, insertion losses in the passbands are relatively high, while the selectivity and band-in-band restraint are unsatisfactory. Moreover, microstrip BPFs using high-temperature superconductors (HTSs) have also been proposed, possessing the merits of the multi-passband design approach, and the insertion losses and band-in-band restraint are significantly improved resulting from the very low surface resistance of the HTS [5]. However, the defects of high cost and strict operation condition are also nonnegligible [6]. Additionally, both the microstrip BPF base polymer and the HTS suffer due to their large size, preventing them from finding application in practical communication systems. To overcome the drawbacks of device size, micro-/nano-fabrication technologies have been applied to develop miniaturized BPFs. Low-temperature co-fired ceramic (LTCC) technology, using the strip lines resonator and the hybrid resonant circuit, is a good candidate for implementing a compact BPF, as a result of its multilayer capability and integrated packing capability. However, such modules possess a low unloaded quality factor (Q-factor), which can be attributed to the poor roll-off and relatively high insertion losses. Meanwhile, the device fabricated by LTCC is relatively thick, resulting from the multilayer manufacturing technology and intrinsic substrate thickness [7,8]. To enhance the Q-factor, high-temperature co-fired ceramic (HTCC) technology, based on a low loss tangent and high dielectric constant material, was employed to design a BPF with the multilayer design approach and rectangular bar step-impedance resonator [9]. A higher Q-factor and good RF performance are achieved by HTCC, but the device size is enlarged as a compromise. In addition to LTCC and HTCC technology, micro-electromechanical systems (MEMS) are also a potential technology for integrating electronic and mechanical components seamlessly into the micro-size chip, with the merits of reduced cost, decreased dimension, and low loss. Nevertheless, devices based on MEMS may suffer from a shortened working life, resulting in uncertainty regarding power handling capability [10]. In addition, some BPFs have been proposed and fabricated using CMOS technology, which can implement devices with smaller overall dimensions, reduced integration costs, shrunk parasitic effects, and low power consumption [11,12]. However, the BPFs constructed using passive components are generally accompanied by drawbacks in the form of electromagnetic interference and eddy currents [13]. Considering these issues, GaAs-based integrated passive device (IPD) technology is fairly attractive for high-performance BPF implementation, as a result of the advantages of high integration, effective cost, and full compatibility [14,15].

In this study, a compact BPF employing GaAs-based IPD technology was developed, comprising two intertwined spiral inductors and a central capacitor. Five air-bridge structures were introduced to the outer quasi-circular inductor with a three-metal-layer design for enhancing the mutual inductive effect [16]. In addition, the intertwined structure is built to construct the differential geometry resulting in the miniaturized overall dimension. After this, a frequency-dependent equivalent circuit under higher frequency excitation was established, and is theoretically analyzed and modeled herein; immediately following this, the 16-step IPD-based fabrication flow of the proposed BPF is explained in detail. The center frequency (CF) of the passband and the transmission zero (TZ) can be shifted by adjusting the turns of the outer inductor and the thicknesses of the top and bottom metal layers. Additionally, a comparison of the BPF constructing the intertwined inductor with/without air-bridges is presented via the simulation result. Finally, an IPD-based BPF featuring a CF operating at 2 GHz and a TZ located at 5.32 GHz was implemented with a miniaturized overall size of 0.037*λ*_0_ × 0.019*λ*_0_ (1537.7 × 800 μm^2^), where *λ*_0_ is the wavelength of the central frequency. It was measured with an insertion loss of 0.38 dB at the CF and 35.15 dB at the TZ, a return loss of 40 dB at the CF, and the 3-dB fraction bandwidth (FBW) of 72.53% after bonding with Au wires and packaged onto a printed circuit board (PCB).

## 2. Materials and Methods 

### 2.1. Design and Circuit Analysis

**Figure 1:**
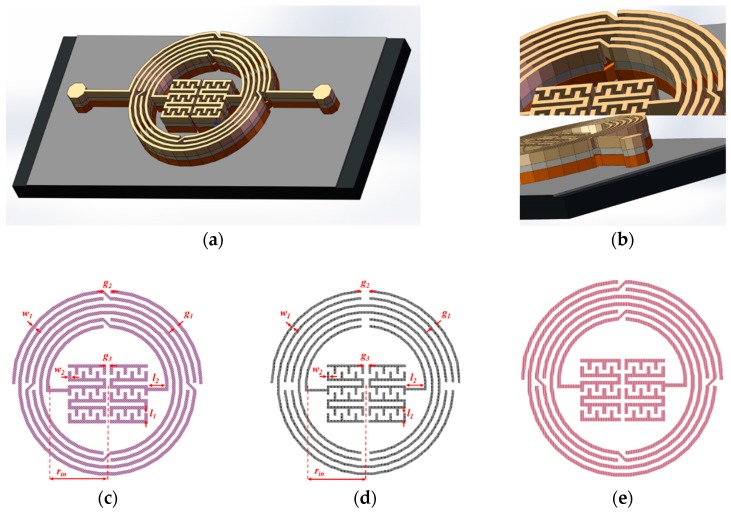
(**a**) 3D view of the proposed BPF; (**b**) enlarged view of the air-bridge structure and side view of the three metal layers; (**c**) leads layer; (**d**) text layer; (**e**) bond layer.

Established with GaAs-based IPD technology, a compact BPF comprising two intertwined quasi-circular inductors and a centrally embedded capacitor was developed with three metal layers, as demonstrated in Figure 1a. Air-bridge structures can be clearly observed in the outer inductor, and an enlarged 3D view of the air-bridge and the three metal layers are shown in Figure 1b. Furthermore, the 2D views of each layer are illustrated in Figure 1c–e, namely: leads, text, and bond from top to bottom in the design and simulation software (Advanced Design System (ADS) 2016). The dimensional parameters of each layer are listed in Table 1, in which the leads and bond layer feature the same dimensions. The width of the conductor coils and the gap between them are set as the minimum manufacturing size to miniaturize the overall size, and the thickness of each layer is also decided by the fabrication. The inner radius is set as 250 μm to embed the central capacitor, and so the distance between the capacitor and the inductor are wide enough to avoid the mutual effects. The number of turns, or the length of the inductor, is estimated via the established equivalent model according to the operating frequency after the inner radius is fixed. 

**Figure 2:**
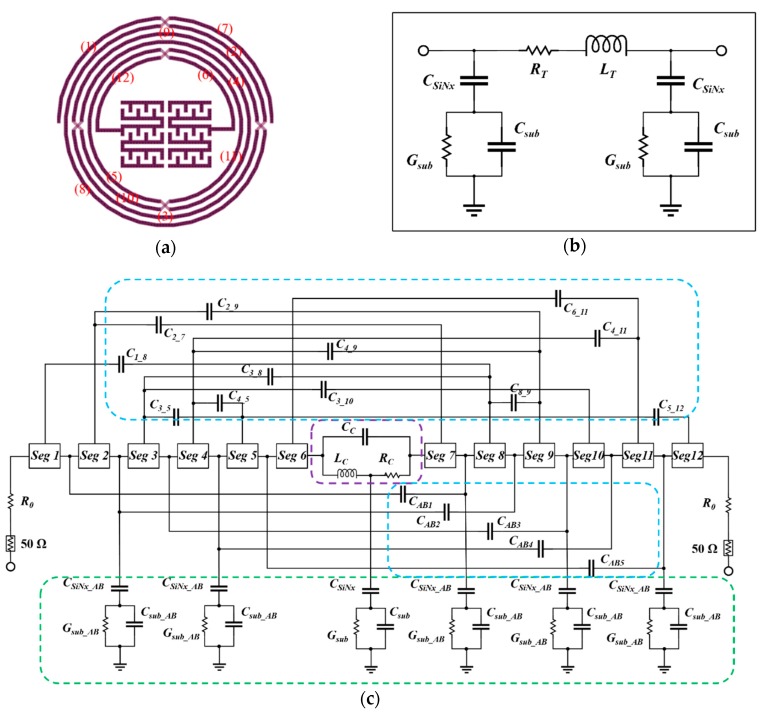
(**a**) Number of segment boxes in the proposed BPF; (**b**) π-type lumped-element model inside the segment box; (**c**) equivalent circuit model of the proposed BPF.

To evaluate the equivalent circuit accurately, an analytical model for estimating the performance during the design process of the proposed BPF was developed based on the segment method, the mutual inductance approach, and simulated scattering parameters (S-parameters) [17,18]. As illustrated in Figure 2a, the lumped model comprises four parts after considering the parasitic effects under high frequency excitation: the segment of the metal line without overlap (*Seg i*), the coupling capacitor between metal tracks out of the segment, the parasitic effects induced by the SiN*x* passivation layer and the GaAs substrate, and the central capacitor.

#### 2.1.1. Model Inside the Segment Box

As shown in Figure 2b, the intertwined inductor is divided into 12 segment boxes by the air-bridge. Each segment box was built by the π-type lumped model, as demonstrated in Figure 2c, where *R_T_* denotes the total series resistance, *L_T_* denotes the total series inductance, *C_SiNx_* denotes the capacitance associated with the SiN*x* passivation layer, and *G_SUB_* and *C_SUB_* denote the conductance and the capacitance associated with the GaAs substrate, respectively. The metal track of each segment box comprises several straight metal lines, and *R_T_* and the *L_T_* are the sums of the resistance and inductance of all the straight metal lines, wherein the effects of the corners can be neglected.

The series resistance and series inductance can be easily evaluated from the metal track geometry and resistivity at a low frequency, in which the current density in the metal track is uniform. However, the spiral inductor under high-frequency excitation possesses nonuniform current distribution in the metal track resulting from the current crowning effect, so the resistance and inductance of each straight metal line is frequency-dependent [19]. Therefore, the concept of the effective linewidth *W_eff_* was proposed to replace the physical metal linewidth for circuit modeling, which can be calculated as the following:

(1) $$W_{eff}=W_{0,i}\left(1-exp\left(-\frac{w}{W_{0,i}}\right)\right),$$

(2) $$W_{0,i}=c1\cdot c2^{i-1}\sqrt{\frac{1}{f}},$$

where *w* represents the physical metal linewidth, *i* represents the turn index, *f* represents the operating frequency, and *c*1 and *c*2 are the fitting parameters for matching the resistance and inductance with the measurement result, respectively, in which *c*1 = 0.669 and *c*2 = 0.56 for the 5-turn spiral inductor. The resistance of each metal line (*R_line_*) for evaluating the losses of the signal can be obtained using the effective linewidth as in Formula (3), which is used as the reference for the trade-off of the cost and deposited metal:

(3) $$R_{line}=\frac{\rho \cdot l}{W_{eff}\cdot t_{m}},$$

where *ρ* is the resistivity (Ω·cm), and *l* and *t_m_* are the length and thickness of the straight metal line, respectively.

The inductance of each segment includes the self-inductance of the straight metal line and the mutual inductance from the other metal track. According to the effective linewidth, the frequency-dependent self-inductance can be expressed as:

(4) $$L_{self}=2l\left(ln\frac{2l}{W_{eff}+t_{m}}-0.5\right).$$

Assuming the metal tracks of each segment are parallel to the others, the mutual inductance can be approximately expressed as:

(5) $$L_{M}=2\times 10^{-4}l\left[ln\left(\frac{l}{d}+\sqrt{1+\frac{l^{2}}{d^{2}}}\right)-\sqrt{1+\frac{l^{2}}{d^{2}}}+\frac{d}{l}\right],$$

where *d* is the distance between the central location of the effective linewidth. Please note that two metal tracks carrying currents in the same direction feature positive mutual inductance, whereas the mutual inductance is negative when the currents flow in the opposite direction [18]. Hence, the total inductance related to the length can be calculated using the formulas (4) and (5), so that the total length of the metal trials can be estimated for the L-band filter, and the length of the air-bridge structures can be determined as well. The components related to the substrate are discussed in Section 2.1.4 together with other components of the same type.

#### 2.1.2. Models Outside the Segment Box

Due to the compact geometry of the spiral inductor, the coupling capacitors between the neighboring metal tracks were introduced and named *C_i_j_*, where *i* and *j* represent the number of the segment box. Such a capacitance can be calculated by employing the approach reported in [20], and the coupling capacitance is given by:

(6) $$C_{i\_j}=\;\varepsilon_{0}\frac{Q}{V},$$

where *Q* denotes the normalized charge and *V* denotes the potential between the central location of two conductors. In addition, the capacitor *C_ABi_*, resulting from the air gap in the air-bridge area between the leads and the bond layer, is considered, and can be determined by:

(7) $$C_{ABi}=\frac{\varepsilon_{0}\left(overlap\;area\right)}{t_{text}}.$$

In combination with the fabrication technology and the calculated length, both capacitances are determined, and can be computed via the Formulas (6) and (7).

#### 2.1.3. Embedded Capacitor

**Figure 3:**
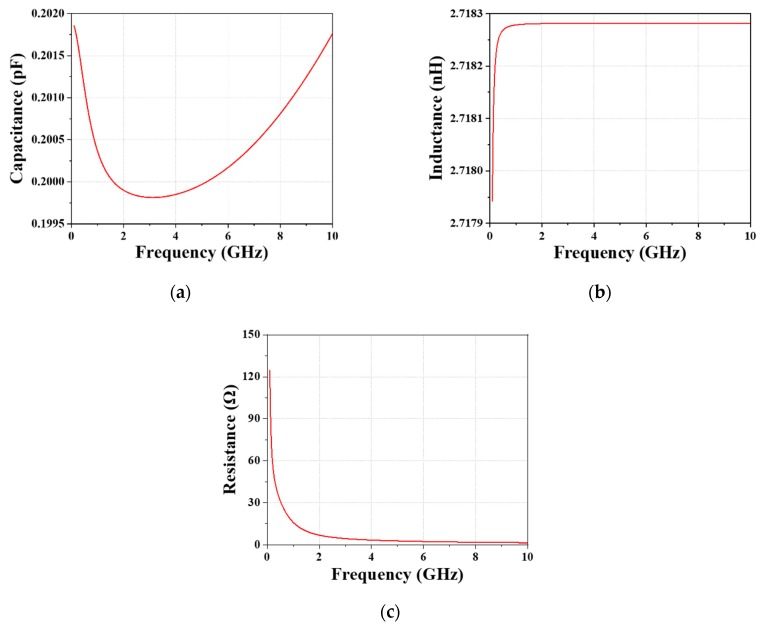
Calculated (**a**) capacitance, (**b**) inductance, and (**c**) resistance of the central capacitor from the Y-parameter in the range of 0–10 GHz.

Considering the ohmic losses and the inductive effect, the central embedded capacitor is modeled by three components: a capacitor (*C_C_*), an inductor (*L_C_*), and a resistor (*R_C_*). However, the frequency-dependent parameters of the capacitor are difficult to calculate due to the complicated construction. Hence, the simulated S-parameters and the Y-parameters are applied to calculate the capacitance, inductance, and the resistance from the given formulas. In addition, the capacitance can be tuned to match the design target via tuning the dimension of the central capacitor. The values of these three parameters of the final designed capacitor varying with frequency in the range of 0–10 GHz are shown in Figure 3. Finally, on the basis of the calculated total inductance and capacitance, the operating frequency can be estimated.

(8) $$C\left(pF\right)=\frac{1\times 10^{12}\times imag\left[Y\left(1,1\right)\right]}{2\pi f},$$

(9) $$L\left(nH\right)=\frac{1\times 10^{9}}{2\pi f\times imag\left[Y\left(1,1\right)\right]},$$

(10) $$R\left(\Omega \right)=real\left[\frac{1}{Y\left(1,1\right)}\right].$$

#### 2.1.4. Parasitic Effects Associated with the Substrate

To realize the accurate design target, the effects of the parasitic inductance and capacitance associated with the substrate and passivation layer should be considered. Three types of components were used to model the parasitic effects arising from the electronic coupling: the capacitor related to the passivation layer (*C_SiNx_* and *C_SiNx_AB_*), and the conductor and capacitor related to the substrate (*G_SUB_*, *G_SUB_AB_*, *C_SUB_*, and *C_SUB_AB_*). The losses resulting from the eddy current are negligible, as the resistivity of the GaAs substrate is larger than 10 Ω·cm [21]. Formulas (11)–(16) are used to compute the above six parameters, and subsequently, the length of the inductor is adjusted to realize the L-band BPF. The components with the subscript *SUB_AB* represent the elements associated with the air-bridge area, which can be considered to be frequency-independent due to their short length. Thus, they can be estimated on the basis of the following equations:

(11) $$C_{SiNx}=\frac{\varepsilon_{0}\varepsilon_{SiNx}\left(air-bridge\;metal\;area\right)}{t_{SiNx}},$$

(12) $$C_{SUB\_AB}=\frac{\varepsilon_{0}\varepsilon_{GaAs}\left(air-bridge\;metal\;area\right)}{t_{GaAs}},$$

(13) $$G_{SUB\_AB}=\frac{air-bridge\;metal\;area}{t_{GaAs}\cdot \rho_{GaAs}}.$$

where *t_SiNx_* and *t_GaAs_* are the thicknesses of the passivation layer and substrate, respectively, and *ε_0_*, *ε_SiNx_*, and *ε_GaAs_* are the permittivity of free space, the passivation layer, and the substrate, respectively. However, the parasitic effects of the capacitor and the metal track of the segment box are frequency-dependent, according to the concept of the effective linewidth. Therefore, a new function *F(W_eff_, t)* with respect to the effective linewidth and metal thickness, and the frequency-dependent permittivity *ε_eff_(f)* were introduced to estimate *C_SiNx_*, *C_SUB_*, and *G_SUB_* as given [22]:

(14) $$C_{SiNx}\left(f\right)=\frac{\varepsilon_{0}\varepsilon_{eff}\left(f\right)}{2F\left(W_{eff},t_{SiNx}\right)}\cdot l,$$

(15) $$C_{SUb}\left(f\right)=\frac{\varepsilon_{0}\varepsilon_{eff}\left(f\right)}{2F\left(W_{eff},t_{SUB}\right)}\cdot l,$$

(16) $$C_{SUb}\left(f\right)=\frac{\sigma_{SUB}\left(1+\sqrt{1+10t_{SUB}/W_{eff}}\right)}{2F\left(W_{eff},t_{SUB}\right)}\cdot l.$$

### 2.2. IPD Fabrication Process

**Figure 4:**
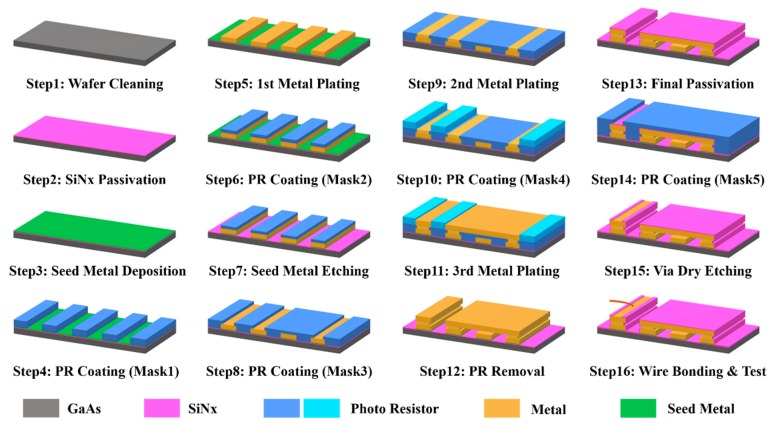
GaAs-based IPD technology fabrication flow.

IPDs are manufactured using standard wafer fabrication techniques with thin-film and photolithography processing, which enables integration of the discrete passive electronic components into a chip [23,24]. An improved IPD fabrication method based on a GaAs substrate was developed in previously reported work by our group. In addition, these components, comprising a NiCr thin-film resistor, spiral inductor, and metal-insulator-metal capacitor, were implemented for the advantages of high quality, high yield, and cost effectiveness [14]. Many blocks, such as the power divider, coupler, and balun, have been developed on the basis of improved manufacturing flow [25,26,27]. The proposed BPF comprising two intertwined spiral inductors and a central embedded capacitor was developed with a total of 16 steps and 5 masks in this study, and the fabrication flow is illustrated in Figure 4.

In step 1, a 6-inch GaAs wafer was prepared as the substrate for the fabrication, and was cleaned using the lift-off machine to spray for 180 s with acetone solution at 23 °C in order to create a flat and clean wafer surface. In addition, the wafer was then polished for 30 s using the HCl solution in the wet station, and blown with N_2_ to dry it. Please note that N_2_ blowing is necessary after each wet process. In step 2, a SiN*x* passivation layer with a thickness of 200 nm was introduced using the plasma-enhanced chemical vapor deposition (PECVD) machine using of SiH_4_, He, N_2_, and NH_3_ as the process gasses. A deposition process of 376 s in the chamber at 250 °C enhances the adhesion of the metal layer and the GaAs wafer. Starting from step 2, a microscope was utilized to inspect for crevices and humps at the end of each step. In step 3, a seed metal layer of Ti/Au with a thickness of 20/80 nm was deposited by sputtering, and the sputtering times of Ti/Au were 50 and 41 s, respectively, with a DC power of 1 KW. In step 4, the negative photoresistor (PR) NR9-3000PY was coated by spin-coating using the coating track with a coating PRM of 1500 and coating time of 40 s and then exposed by the contact aligner using mask 1 to define the pattern of the first metal layer. Then, following inspection with the microscope, the critical dimension scanning electron microscope (CD-SEM) was introduced to check the pattern of the design after PR was exposed. Subsequently, the first metal layer of Cu/Au with the thickness of 4.5/0.5 μm was generated by electroplating with a current of 300/70 mA and a processing time of 17/4 min, respectively, and the PR was removed with acetone solution using a lift-off machine immediately after step 5. In step 6, the PR was coated with a coating PRM of 1500 and coating time of 40 s, and exposed with mask 2 to protect the plated first metal layer in the following etching process. In step 7, the needless seed metal was etched by SF_6_/Ar plasma using an inductively coupled plasma (ICP) etcher with a gas flow rate of 20 sccm and process time of 375 s. Subsequently, after microscope inspection, a DC check was employed to confirm that the unwanted area had been etched, and immediately after this, the thickness was measured with the surface scan profiler. Next, in step 8, the negative PR was applied with mask 3 to define the structure of the second metal layer, while the gap of the air-bridge between the third and first metal layer was defined. Then, in step 9, Cu with a thickness of 1.8 μm was electroplated with a current of 300 mA and a processing time of 7 min for the second metal layer. In step 10, a PR process based on mask 4 was adopted using positive PR AZ7220, and in step 11, the third metal layer was electroplated with the same thickness and material as that of the first metal layer. Subsequently, in step 12, the PR was manually stripped off via wet etching and acetone solution with a spray time of 300 s, and the intertwined coils and central capacitor were implemented with the completion of this step. To protect the device from moisture and oxidation, in step 13, a final passivation layer using 300 nm SiN*x* was deposited through the PECVD with a process time of 564 s. In step 14, the PR process was adopted with mask 5 to generate an open area for via etching. Next, in step 15, the ICP dry etching process using SF_6_/O_2_ with a gas flow rate of 45/5 sccm and processing time of 120 s was employed to generate the via in the defined area for the following wire-bonding, then, the PR was removed using acetone with a spray time of 180 s. Finally, in step 16, polishing, dicing, and wire-bonding processes were employed, enabling the RF performance of the fabricated device to be tested. Table 2 lists the manufacturing techniques used in the fabrication process.

## 3. Discussion and Results

### 3.1. Effects of Variable Dimension Parameters

**Figure 5:**
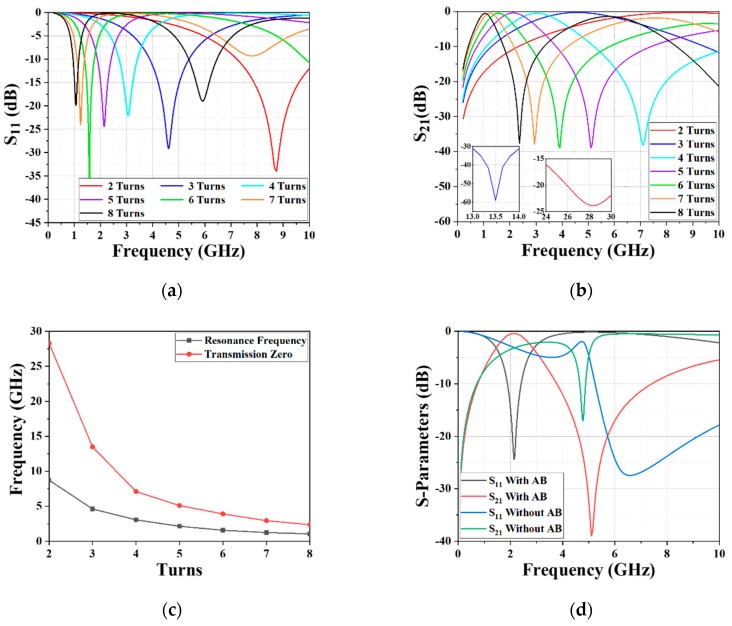
Effect of the varying inductor turns on (**a**) S11 and (**b**) S21; (**c**) functional relationship between the CF and the number of turns; (**d**) S-parameter comparison of the intertwined inductor with/without air-bridge structure.

Factors such as the overall size, insertion loss, and bandwidth are important for the performance evaluation of a BPF, and the controllability of the CF and TZ is an additional factor in evaluating a BPF device. Our previous work reported the impact of the varying inner radius, conductor gap, and conductor width [28]. To further investigate the controllability of the proposed IPD-based BPF, the effects of the altering turns of the outer inductor and various metal thicknesses on the RF performance were analyzed via the simulation software. Figure 5a,b illustrates the trails of the reflection coefficient (S11 parameter) and transmission coefficient (S21 parameter) with varying inductor turns in the range of 2–8, respectively. It can be obviously noted from Figure 5a that the CF shifted to a lower frequency with the increasing inductor turns; the TZ presents the same shifting trend as the CF, as revealed in Figure 5b. The functional relationship of the CF and the number of turns is shown in Figure 5c, indicating that the CF increases exponentially as the number of turns decreases, rather than having a linear relationship. In addition, the comparison of the intertwined inductor with/without air-bridge structure is illustrated in Figure 5d, demonstrating that differential construction with air-bridge structure improves the performance in the L-band and S-band.

**Figure 6:**
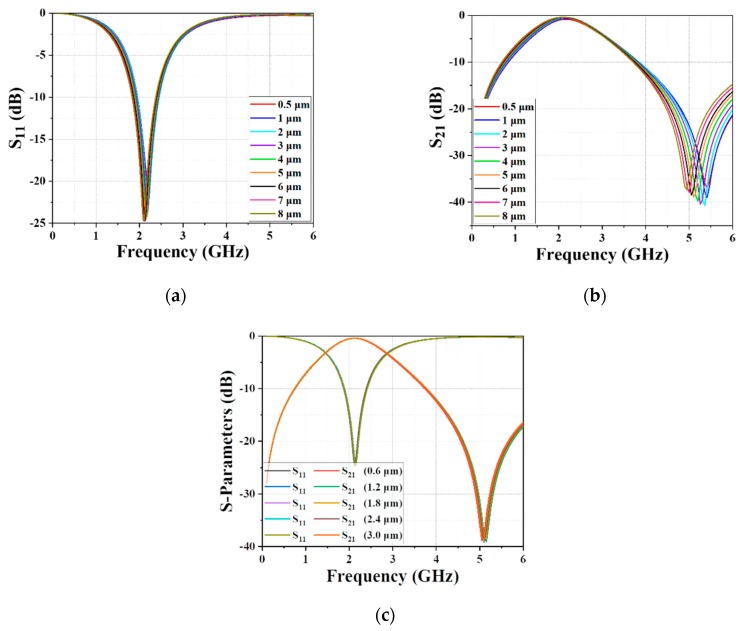
The effect of varying the metal thickness of the leads and bond layer on (**a**) S11 and (**b**) S21; (**c**) the effect of varying the metal thickness of the text layer.

Figure 6 shows the variation caused by altering the metal thicknesses of the different layers, in which the leads and the bond layer always have the same thickness. Figure 6a demonstrates the effects of the varying metal thicknesses of the leads and bond layer on the S11 parameter when the metal thickness of the middle layer was fixed at 1.8 μm, indicating that the CF barely changed with the increasing metal thickness in the range of 0.5–8 μm. However, the insertion loss at the CF increased when the metal thickness was lower than 2 μm, which is interpreted as the strengthened resistance resulting from the metal thickness being lower than the skin depth of copper at 2 GHz. The skin depth of copper is approximately equivalent to 1.48 μm, as can be calculated from Equation (17):

(17) $$d=\frac{1}{\sqrt{\pi f\mu \sigma }},$$

where *f* denotes the operating frequency, *μ* denotes the permeability, and *σ* denotes the conductivity. Figure 6b reveals the relationship between the S21 parameter and the metal thickness, indicating that the TZ moved to higher frequency with the increasing metal thickness. The effect of the metal thickness of the text layer on the S-parameters is given in Figure 6c, wherein the metal thickness of the text layer was adjusted from 0.6 μm to 3 μm, while the other two layers were set as 5 μm. No evident changes can be noticed, indicating that the middle metal layer barely affects the RF performance.

### 3.2. Results

**Figure 7:**
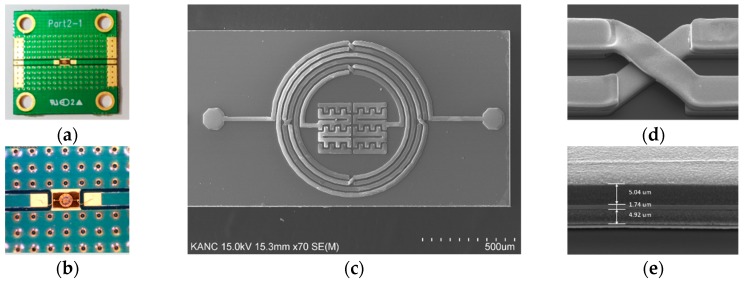
(**a**) Packaged BPF on the printed circuit board; (**b**) enlarged view of the wire-bonded BPF; (**c**) SEM figure of the top view of the BPF; (**d**) enlarged view of the air-bridge area; (**e**) cross-section of the device using the GaAs-based IPD technology.

**Figure 8:**
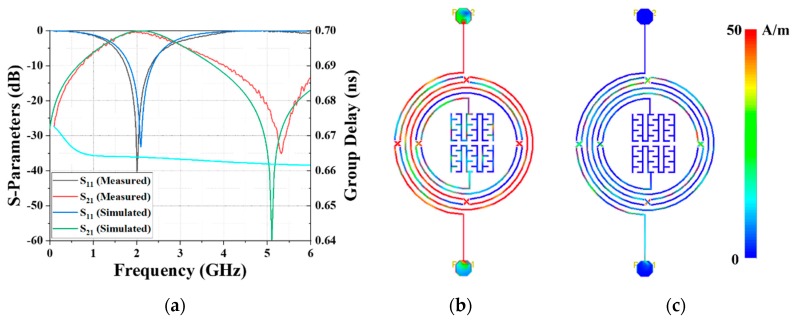
(**a**) Comparison of the measured and simulated results; current density at (**b**) 2.13 GHz and (**c**) 5.31 GHz.

After being simulated and optimized by the ADS 2016 software, the proposed BPF was fabricated based on the IPD technique on the GaAs substrate with a thickness of 200 nm, a dielectric constant of 12.85, and a tangent loss of 0.006, to verify the design approach. The device possesses a miniaturized overall size of 0.037*λ*_0_ × 0.019*λ*_0_ (1537.7 × 800 μm^2^), then it was bonded with Au wires and packaged onto a 2 cm × 2 cm PCB for convenience of performance characterization, as shown in Figure 7a. Figure 7b shows a magnified image of the packaged BPF taken by a Nikon Eclipse L150 microscope, and the SEM figure of the top view is demonstrated in Figure 7c. Figure 7d provides an enlarged area of the air-bridge structure, and depicts the narrow text layer in the transmission line area and the gap between the leads and bond layer in the cross area. Figure 7e illustrates the cross-section of the IPD-based device, in which the three metal layers with different thicknesses and the bottom SiN*x* passivation layer can be evidently observed. The Agilent 8510C vector network analyzer (VNA) was applied to measure and record the S-parameters. The simulated and measured S-parameters of the proposed BPF are compared and presented in Figure 8a, showing that good agreement was achieved. The frequency bias of the CF and TZ is due to the fabrication tolerance and the effect of the wire-bonding and package process.

As depicted in Figure 8a, the CF of the simulated and measured results operates at 2.09 GHz and 2 GHz, respectively, and a TZ is located at the right side of the passband with the frequency of 5.1 GHz and 5.32 GHz, respectively. The insertion loss at the CF and TZ of the measured results are 0.38 dB and 35.15 dB, respectively, achieving a low loss pass in the passband and good out-of-band isolation. Additionally, the group delay is also simulated, and is shown in Figure 8a [29,30]. The current density distribution at the CF and TZ are shown in Figure 8b,c, validating the excellent passband performance and out-of-band restraint. Moreover, the implemented BPF features an ultrawide passband from 1.387 GHz to 2.822 GHz with a 3-dB FBW of 72.53%. As a significant figure of merit (FoM) of BPF, the quality factor is calculated using the simulated Y-Parameters as 32.2 [31,32,33]. The performance comparison of the proposed IPD-based BPF with recently published work, based on various manufacturing technologies, is given in Table 3, which exhibits the advantages of the low insertion loss, wide passband, and miniaturized size of the GaAs-based IPD technology. Furthermore, the comparison of this work GaAs-based IPD with other BPF using different GaAs-based technologies is shown in Table 4, showing the IPD-based device possess the merits of ultra-wide passband and low insertion loss with similar dimension.

## 4. Conclusions

A microscale BPF comprising two intertwined spiral inductors and a centrally embedded capacitor was developed using GaAs-based IPD technology in this study. The equivalent circuits model was produced after considering the capacitive and inductive parasitic effects using the segment method, mutual inductance approach, and simulated S-parameters. The three-layer IPD manufacture flow with the thin-film and photolithography processes were presented in 16 steps. The fabricated BPF features a miniaturized overall size of 0.037*λ*_0_ × 0.019*λ*_0_ (1537.7 × 800 μm^2^). Furthermore, the analysis of the varying dimensional parameters reveals the relationship between the S-parameters and the BPF size. Finally, the measured results show a good consistency with the theoretical prediction and simulation, demonstrating a low insertion loss of 0.38 dB, 10-dB out-of-band suppression to 6 GHz, and an ultrawide 3-dB FBW of 72.53%. The proposed BPF could be an excellent candidate for a modern communication system due to the high performance and miniaturized size, and the GaAs-based IPD technology used for fabrication present the great potential to manufacture miniaturized passive devices. However, lifecycles and compatibility of the practical application were not discussed in this paper, and the selectivity is unprovable, which will be the focus of future work in promoting the practical application of IPD technology.

## Figures and Tables

**Table 1:** Dimensional parameters of leads, bond, and text layers

Parameter (mm)	*w1*	*w2*	*g1*	*g2*	*g3*	*r_in_*	*l*_1_	*l*_2_	Thickness
Leads and Bond	15	15	15	30	15	250	75	30	5
Text	11	11	19	34	19	252	79	30	1.8

**Table 2:** Fabrication techniques and materials used in the manufacturing process.

Fabrication Objective	Technique	Material	Application of Step
Passivation layer	PECVD	SiN*x*	2, 13
Photoresistor	Spin-coating	Negative/positive PR	4, 6, 8, 10, 14
PR removal	Lift-off	Acetone	5, 12
Seed metal	Sputtering	Ti/Au	3
Metal layer	Electroplating	Cu/Au	5, 9, 10
Via	ICP etching	SF_6_/O_6_	7, 15

**Table 3:** Comparisons with other works using various manufacturing technologies

Ref.	Technique	CF (GHz)	IL (dB)	RL (dB)	FBW (%)	Circuit Area
[34]	Microstrip	0.975	1.1	>20	13.3	0.094*λ*_0_ × 0.08*λ*_0_
[35]	HTS	1.9/2.6	0.18/0.32	>16	2.6/2.4	0.182*λ*_0_ × 0.156*λ*_0_
[36]	LTCC	2.4	2.4	15	12.5	0.058*λ*_0_ × 0.058*λ*_0_
[9]	HTCC	2.25	1.8	>15	5.5	6.9 × 39.9 mm^2^
[37]	Si-based IPD	1.7	2.54	12	16	2.6 × 1.5 mm^2^
This work	GaAs-based IPD	2	0.38	40	72.53	0.037*λ*_0_ × 0.019*λ*_0_ (1.54 × 0.8 mm^2^)

**Table 4:** Comparisons with other works using GaAs-based technology

Ref.	Technique	CF (GHz)	IL (dB)	RL (dB)	FBW (%)	Circuit Area
[38]	IPD	3.2/5.8	1.2/0.6	22/25	7.8/33.3	0.2*λ*_0_ × 0.11*λ*_0_
[39]	HBT	3.35	2.05	19	55	0.02*λ*_0_ × 0.05*λ*_0_
[40]	MMIC	3.01	5.6	7.5	-	0.7 × 0.4 mm^2^
[41]	SIW	93	4.3	>13.5	3.4	2.31*λ*_0_ × 1.57*λ*_0_
This work	IPD	2	0.38	40	72.53	0.037*λ*_0_ × 0.019*λ*_0_ (1.54 × 0.8 mm^2^)
